# Supplementary material for: Adaptive Evolution for the Efficient Production of High-Quality d-Lactic Acid Using Engineered Klebsiella pneumoniae
Source: Microorganisms. 2024 Jun 8;12(6):1167. doi: 10.3390/microorganisms12061167 (PMC11205318; doi:10.3390/microorganisms12061167)
Supplement: Supplementary file 1 [file microorganisms-12-01167-s001.zip › Supplementary Table.pdf]

Supplementary Table S1 Primers used in this study

Primers	Nucleotide Sequence (5’-3’)
**Construction of *Klebsiella pneumonia* Δ*rcsA***	
3253	CGAGCTCTTAGCAAGTTTGATCCCGACTC
3254	GGATGGGTCATGTCAACGAACAAACTGGCGG CGCTC
3255	GAGCGCCGCCAGTTTGTTCGTTGACATGACCC ATCC
3256	GGGGTACCCTCACTGTCACGATTCATCAG
3257	AGTCACCAGGCGCCAACATTC
3258	GTGAGACGTTCAACACGGGAC
**Construction of *Klebsiella pneumonia* Δ*galU*,**	
3265	CGAGCTCCACACGCGCAGGCGATAATG
3266	CTTTCAACCGCGCGGCGGACCTGGTGCCTAAT CCCGCAA
3267	TTGCGGGATTAGGCACCAGGTCCGCCGCGCGG TTGAAAG
3268	GCTCTAGATGAAAGATGTGAAGCGGCAC
3269	CACTGCCGTATTCATTATCGAC
3270	CTCTCAAAAAGCATACCGTCTC
**Construction of *Klebsiella pneumonia* Δ*ydhS***	
3271	CGAGCTCTCTCCATCGCCAGCGAAGAG
3272	TTGCGCTTAAGCGCTCCGGATTCTCTCCCTTCCTGT

3273	ACAGGAAGGGAGAGAATCCGGAGCGCTTAAGCGCAA
3274	GGGGTACCGAGAGAAACTGGTGCAGCAG
3275	GTTCAAACAGCAGCCCATAG
3276	TTCCAGCGGATAGCGCATTC
1591	CCATATCACCACGTCAAAGGGT
1593	CAAGATGTGGCGTGTTACGGT

Construction of pACYCduet1$_{\triangle T7/plac1\text{-}6}$ -*galU*

3261	CGGGATCCGATGCTGCCGGCCACCAAAG
3262	CGAGCTCTTACTTCGCTACCGCCGTTTC
140	GATTATGCGGCCGTGTACAA
142	GGATCTCGACGCTCTCCCT

Construction of *Klebsiella pneumonia* A1Δ*adhE*Δ*ackA*Δ*budB*, *Klebsiella pneumonia* A4Δ*adhE*Δ*ackA*Δ*budB*, *Klebsiella pneumonia* B1Δ*adhE*Δ*ackA*Δ*budB*

1381	GCTCTAGAATGGCTGTTACTAATATCGC
1382	CGACGCCGATAGCAGGTTTAC
1383	GTAAACCTGCTATCGGCGTCGGTGCGTTCGGTGGTCTGGAT
1384	GGGGTACCTCAGCCTTTACCGGAGCAAC
1385	ATAATGTCGAATCGAGCGAC
1386	GCTTGTCGCGATGCTATCGC
1387	GCTCTAGAATGTCGAGTAAGTTAGTAC

1388	GCGTAGAGATAGGATTCTTC
1389	GAAGAATCCTATCTCTACGCGAAGGCCTGGTG ATGGGTAC
1390	CGAGCTCTTATGCGGTCAGACGGCTGGC
1391	ATCCTGCGCTACGCTAATGAC
1392	CCTGCAGCTCGAATTATTGC
1448	GCTCTAGAGTCAGTCAGCTGGAAGCTC
1449	ATAAGCTTCGCCACCTGGTC
1450	GACCAGGTGGCGAAGCTTATCTGCGCATCGTT CGCGCCAT
1451	CGAGCTCTTATCGCGATAATCTACCG
1452	ATGGACAAACAGTATCCG
1453	ACAGAATCTGACTCAGATG

Reverse transcription PCR

rpoD

3243	TGATGGATCGCGTTCGTACTC
3244	TGAACCAGGTTTCGCTGGTTT

23s rDNA

3245	AAAGAAATCAACCGAGATTCCC
3246	CAGACCGTTCCACTAACACACAA

rcsA

3239	TATGCTGCAAATGTGGATGGC

3240	CGGAGGTGATGTTTTCGGTC
ydeD	
3241	GCTGGCCTTGCAGGTTTCTG
3242	GAAGGTGTCCCCTTCTTTGGTG
galU	
3509	CCACCAAAGCGATTCCAAA
3510	TCCTGCAGCGATACATTCA

Supplementary Table S2 Gene characteristics of *K. pneumoniae* (ATCC25955)

Genome	Genome Size	Total Number	Total Length	Length/Genome Length(%)	GC Content(%)
K. pneumoniae (ATCC25955)	5,339,625	5,076	4,657,851	87.23	58.64

Supplementary Table S3 ncRNA characteristics of *K. pneumoniae* (ATCC25955)

Type	Copy Number	Total Length(bp)	Genome Content(%)
tRNA	86	6,743	0.1263
5s rRNA	9	1,035	0.0193
16s rRNA	8	12,224	0.2289
23s rRNA	8	23,200	0.4344
sRNA	53	4,429	0.0829
