# Supplementary figures and images for: Adaptive Evolution for the Efficient Production of High-Quality d-Lactic Acid Using Engineered Klebsiella pneumoniae
Source: Microorganisms. 2024 Jun 8;12(6):1167. doi: 10.3390/microorganisms12061167 (PMC11205318; doi:10.3390/microorganisms12061167)

## Supplementary Figures

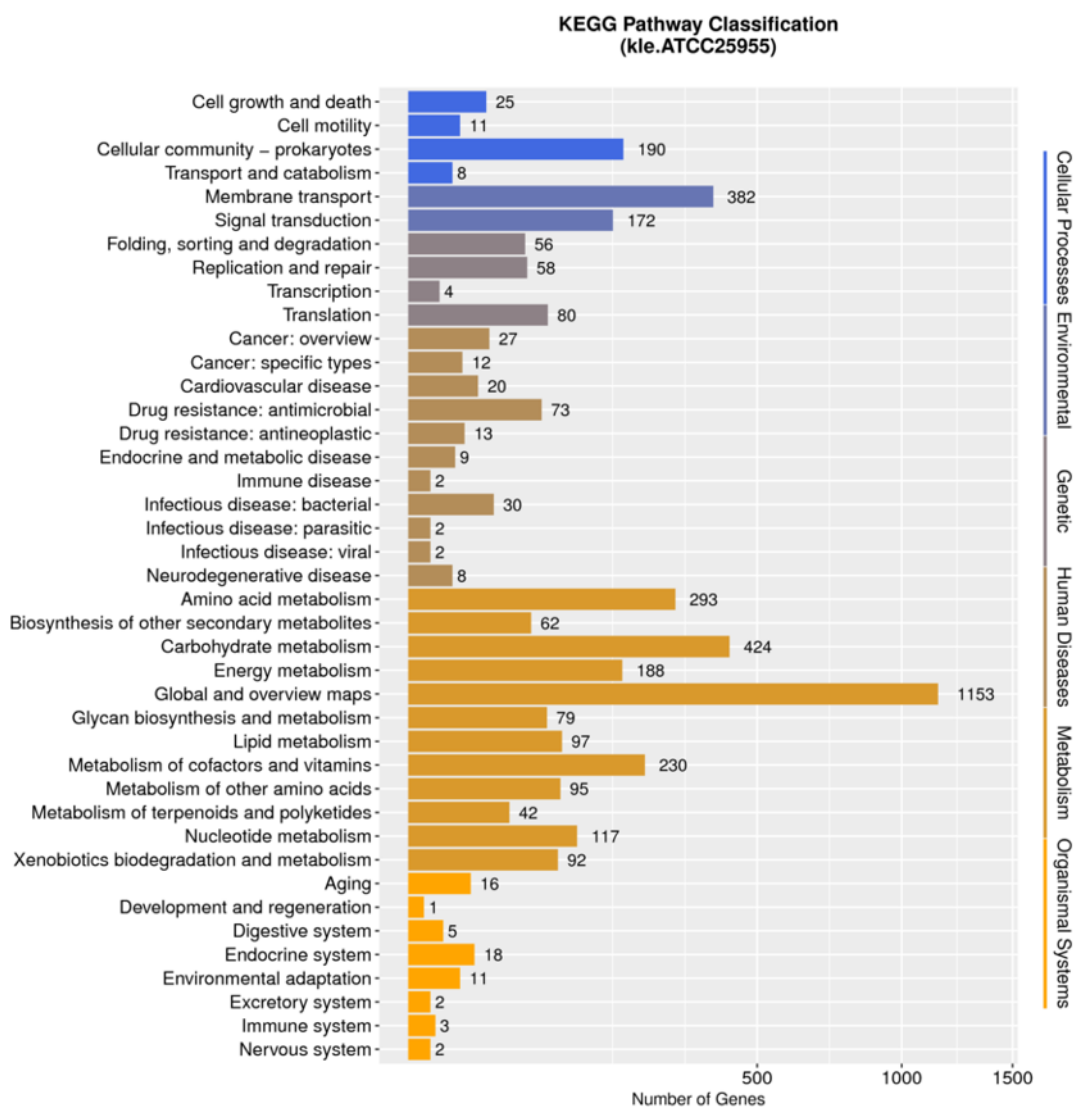

Figure S1.

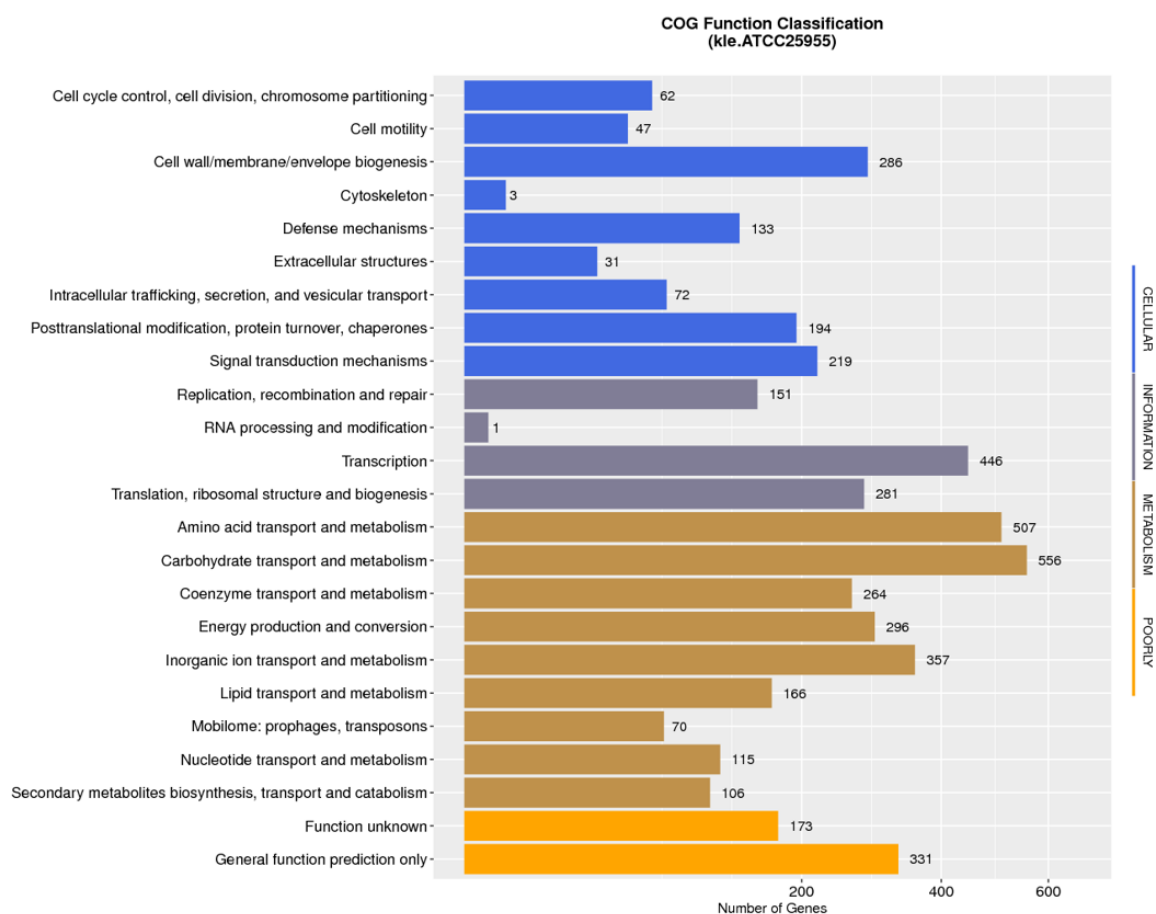

**Figure S2.**

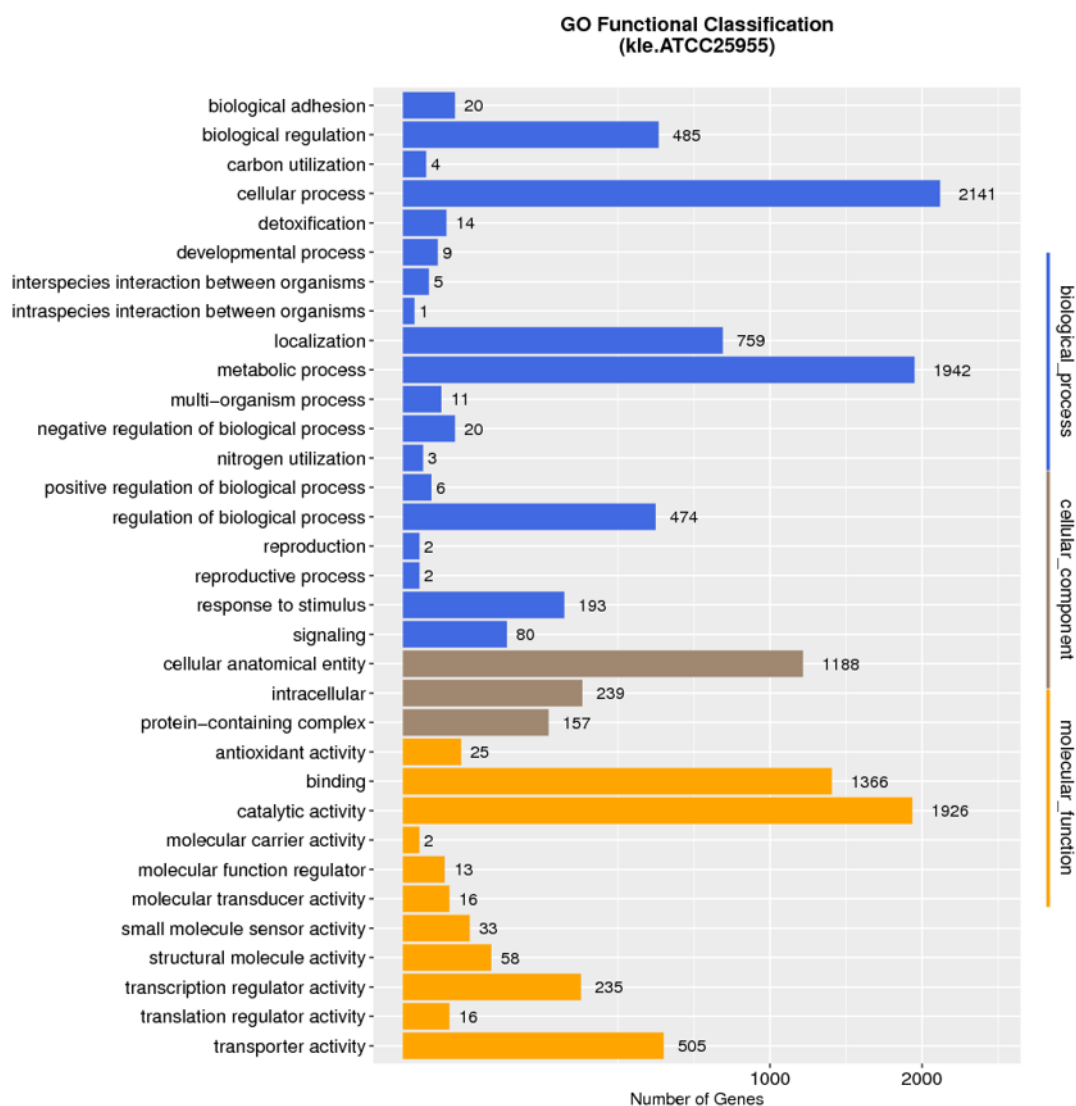

Figure S3.

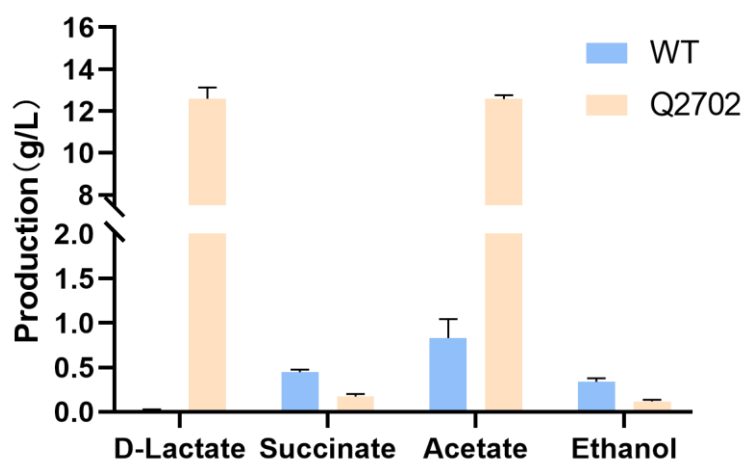

Figure S4.

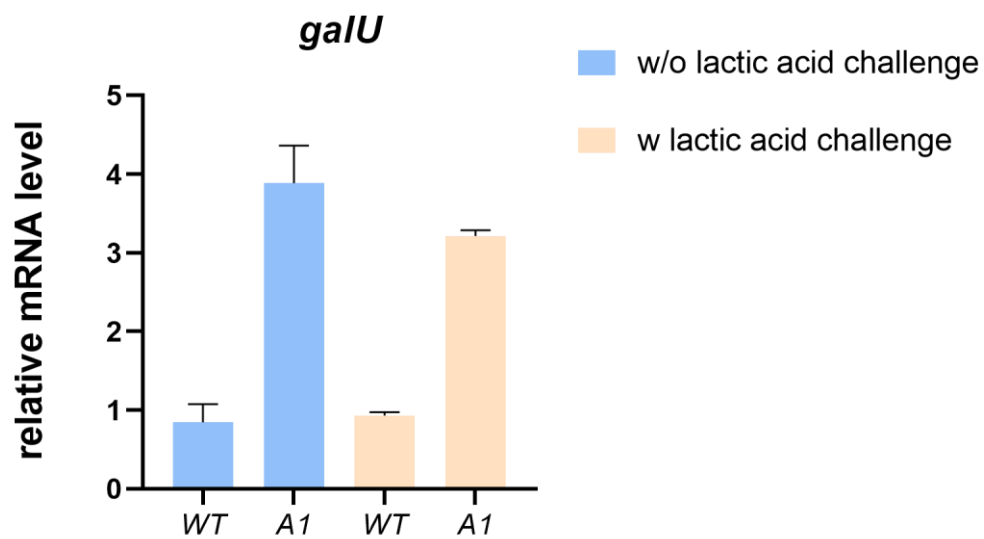

Figure S5.

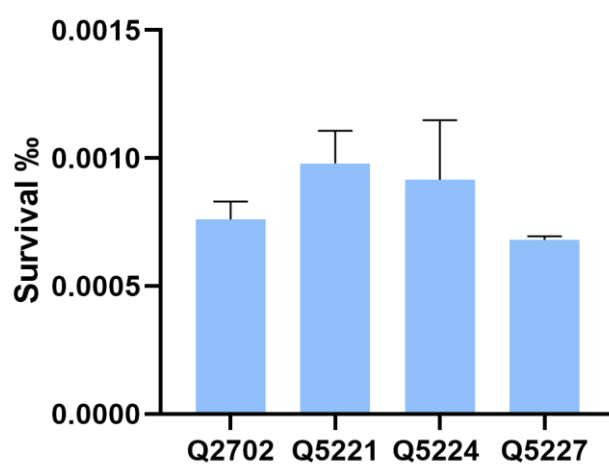

Figure S6.

Supplement: Supplementary file 1 [file microorganisms-12-01167-s001.zip › Supplementary Figures.pdf]
